# Radiobiology of Radiosurgery for the Central Nervous System

**DOI:** 10.1155/2013/362761

**Published:** 2013-12-29

**Authors:** Antonio Santacroce, Marcel A. Kamp, Wilfried Budach, Daniel Hänggi

**Affiliations:** ^1^Departments of Radiation Oncology and Neurosurgery, Medical Faculty, Heinrich Heine University, Moorenstraße 5, 40225 Düsseldorf, Germany; ^2^Department of Neurosurgery, Medical Faculty, Heinrich Heine University, Moorenstraße 5, 40225 Düsseldorf, Germany; ^3^Department of Radiation Oncology, Medical Faculty, Heinrich Heine University, Moorenstraße 5, 40225 Düsseldorf, Germany

## Abstract

According to Leksell radiosurgery is defined as “*the delivery of a single, high dose of irradiation to a small and critically located intracranial volume through the intact skull.*” Before its birth in the early 60s and its introduction in clinical therapeutic protocols in late the 80s dose application in radiation therapy of the brain for benign and malignant lesions was based on the administration of cumulative dose into a variable number of fractions. The rationale of dose fractionation is to lessen the risk of injury of normal tissue surrounding the target volume. Radiobiological studies of cell culture lines of malignant tumors and clinical experience with patients treated with conventional fractionated radiotherapy helped establishing this radiobiological principle. Radiosurgery provides a single high dose of radiation which translates into a specific toxic radiobiological response. Radiobiological investigations to study the effect of high dose focused radiation on the central nervous system began in late the 50s. It is well known currently that radiobiological principles applied for dose fractionation are not reproducible when single high dose of ionizing radiation is delivered. A review of the literature about radiobiology of radiosurgery for the central nervous system is presented.

## 1. Introduction

Over the last century radiation therapy for the management of human cancer had a tremendous evolution. Radiation delivery is based in most cases on photon irradiation. It might be applied with different techniques devices and dose schedules. More recently in particular at the beginning of this century experiments were performed with heavier ions radiation such as protons or carbon ions; in more recent times this highly biological effective radiation was also introduced in clinical routine thus with encouraging results but deserving longer term followup to draw better conclusions and therefore to establish clinical therapeutic indications.

The central nervous system (CNS), in particular the brain parenchyma, deserves some separate considerations [1]. First the TNM classification cannot be applied. Furthermore secondary metastases of primary brain tumors even if malignant are a truly rare event, described in the literature, but nevertheless uncommon [2].

Therefore it is not surprising that CNS may react differently when exposed to ionizing radiation compared to other organs of the human body. This makes the CNS somehow “unique” in the field of radiation oncology, radiobiology, and radioresistance.

Any physician is aware of the eloquence of specific nervous regions, like speech areas, optic pathways, brainstem, limbic lobe, and the “respect” they deserve. Radiation therapy is not an exception to this issue.

Assuming these presumptions Larsson et al. began radiobiological investigations to study the effect of high dose focused radiation on the central nervous system more than 5 decades ago. The introduction of highly focused single session radiation delivered with the help of stereotactic framed based coordinates within the brain led to the definition of radiosurgery [3, 4].

Today it is not surprising, assuming these considerations, that the radiobiological principles used for dose fractionation are not applicable for single session radiation most particularly for the CNS. These controversies come out either from experimental data or from clinical experience [1, 5].

Together with radiosurgery as neurosurgical tool, newly developed high precision radiotherapy techniques, that is, stereotactic fractionated radiation and intensity modulated radiotherapy (IMRT), are now steadily establishing their role in definitive cancer therapy. While advantages of the new radiation techniques are evident upon physical grounds a few radiobiological issues remain unresolved regarding the evaluation of radiation doses employed in these treatment modalities [6].

First aim of this paper is to review the studies currently published about radiobiological principles for radiosurgery, clarifying definitions and terminology used for stereotactic radiation techniques.

Second aim is to review and compare the reliability of the mathematical formalisms used in clinical dose fractionated radiotherapy when applied to radiosurgery.

Third aim is a brief summary of the clinical indications and experimental lines of evidence of radiosurgery for the central nervous system.

## 2. Radiosurgery and Fractionated Stereotactic Radiotherapy

The use of “stereotactic coordinates” implies the support of three-dimensional mapping techniques to perform a medical procedure which might be applied to radiation therapy and surgery in particular of the central nervous system. Stereotactic radiation is a highly precise technique to deliver conformal radiation to a small target volume, either neoplastic or nonneoplastic sparing surrounding tissue by radiation exposure. This radiation if applied as high single dose fraction, in most of cases of photons, is defined as radiosurgery (RS), also named by many authors as stereotactic radiosurgery (SRS). If this radiation dose is delivered using more than one dose fraction, always with support of stereotactic coordinates, it is defined as fractionated stereotactic radiotherapy (FSRT). Stereotactic fractionated radiotherapy should not be confused with intensity modulated radiation therapy (IMRT). In this case a cumulative radiation dose is, like for 3D conformal radiation, applied to a given target volume with dose fractionation but without supply of stereotactic coordinates. Furthermore individual treatment beams irradiate only part of the target at the time: it assigns nonuniform intensities (i.e., weights) to tiny subdivision of irradiation beams defined as “beamlets.”

The current radiosurgery concept is that damage to tissue within the target volume (either normal or neoplastic) is the desired effect. Historically developed by Larsson et al. [3], the number of clinical indications of radiosurgery has increased greatly. Nowadays radiosurgery has a well-established role for the treatment of small volume brain lesions like AVMs [7, 8], vestibular schwannomas [9], small remnants recurrent WHO Gr. I meningiomas, and imaging defined meningiomas [10–13] and in more recent times also in the field of functional disorders such as trigeminal neuralgia and pharmacological resistant epilepsies.

## 3. Radiobiological Principles

### 3.1. 4Rs of Radiobiology

The goal of any medical intervention is to reach the highest rate of clinical success in terms of desired effect with the minimum rate of side effects treatment related.

The radiobiological (toxic) effect obtained with radiation therapy aims to achieve a high tumor control rate together with low complications rate treatment related. Historically radiation was delivered considering variable safety margin to include microscopic tumor infiltration in normal tissue.

Dose application, in particular for malignant brain lesions, consisted in the application of cumulative dose into a variable number of fractions, usually not more than 2 Gy for standard fraction more than 2 Gy for hypofractionation either in curative or in palliative setting. The cumulative dose was currently applied with 3D imaging simulation obtained with CT scan and MRI support. The relationship between time of radiation, dose, and number of fractions to influence biological effect to a given tissue is based on four basic principles of radiobiology defined as the “4Rs” of ionizing radiation [14, 15]:

1. *repair* capacity of cells after sublethal damage radiation induced,
2. *repopulation* of surviving tumor stem cells during fractionated radiotherapy,
3. *redistribution* of cells between the cell cycle after radiation injury in equally distributed radiation sensitive and resistant subpopulations,
4. *reoxygenation* of hypoxic tumor cells after repeated radiation exposure. The radiosensitivity of cells is inversely proportional to the hypoxic cell rate. The application of a dose fraction produces death of oxygenated tumor cells followed by oxygenation of hypoxic cells now more sensitive to the following dose fraction.

These radiobiological principles are based on “in vitro” experiments applying a dose of ionizing radiation, usually photons, to cell in vitro cultivated, for example, fibroblasts [14, 15]. The results of these experimental models together with clinical experience with three-dimensional conformal radiotherapy brought the physicians to conclude that dose fractionation lessens the risk of injury of normal tissues and thus the rate of side effects [16].

It has been reported that the relationship between radiation dose to desired outcome and undesired effect can be represented by a sigmoid dose-response curve [17]. The logical consequence of this sigmoid curve representation is to define a therapeutic window through which the better desired outcome can be achieved (i.e., imaging control of the target volume) without increasing the rate of undesired outcomes (complication rate). Reducing the volume of tissue irradiated shifts dose-response curve for complications increasing the separation between cure and complication probability [17].

The principle underlying radiosurgery is that by reducing the volume target and the safety margin applied with a high conformal radiation delivery the complication rate might be drastically lower if compared to larger target volumes.

According to this presumptions the following definitions of radiosurgery are available:

1. “the delivery of a single high dose of irradiation to a small and critically located intracranial volume through the intact skull” [3],
2. “stereotactic radiosurgery: stereotactically guided delivery of focused radiation to a defined target volume in single session” [17],
3. “discipline that uses externally generated ionizing radiation delivered in single session to eradicate or inactivate a target defined by high resolution stereotactic imaging” (ASTRO SRS Model Coverage Policy),
4. “technique designed to deliver a high dose of focused radiation to a defined target volume to elicit a decide radiobiological response” [18].

Goals of Radiosurgery are as follows:

1. exposure of a target volume to a single high dose of ionizing radiation which ultimately translates into a specific (toxic) radiobiological response [17],
2. precise destruction of a chosen target containing healthy and/or pathological cells, without significant concomitant or late radiation damage to adjacent tissue [3].

### 3.2. Mathematical Formalisms and Models

*The Linear Quadratic Model*. The biological response of a given tissue to radiation and dose fractionation has been theorized with many models. The linear quadratic (LQ) equation formula is so far the most used: briefly it defines the biological response of tissue in terms of surviving fraction (SF) to a given dose (*D*) according to a linear dose coefficient *α* for low doses and a coefficient for the square of the dose *β* for high dose fraction within a dose range from 1 untill 8 Gy; biological effect is proportional to *αD* + *βD* ^2^:

(1) $$\begin{array}{c} \text{SF}=e^{-(a*D+\beta *D^{2})}; \end{array}$$

then

(2) $$\begin{array}{c} \ln\text{SF}=-\alpha D-\beta D^{2}. \end{array}$$

The extrapolated cellular survival curve related to the dose applied allows us to define a linear component of cell killing *α* and a quadratic component of cell killing *β*. The ratio of these two variables defines *α*/*β* ratio of a tissue and expresses the point where the linear component *α* and the exponential (curvier) component of the survival curve *β* are equal or, in other words, it express the dose at which the two components of cell killing are equal.

The linear quadratic formula is presently the standard way to mathematically represent the effect of radiotherapy to account for the effects of different fractionation schedules [5].

The clinical consequence of this model is that normal tissues can be classified in *early* responding tissue (high *α*/*β* ratio) and *late* responding tissue (low *α*/*β* ratio). For neoplastic tissues this classification reports relatively high *α*/*β* ratios for malignant tumors and low *α*/*β* ratios for slow growing benign tumors.

Rationale of dose fractionation is to reach a compromise between desired effect (tumors cure/target destruction) and undesired effects (injury of normal tissue/complications): by delivering a cumulative dose in several fractions it is possible to spare normal tissue from severe damage and to repair from sublethal damage. Moreover the reoxygenation of hypoxic tumor cells after repeated radiation exposure reduces the radioresistance of the tumor tissue thus increasing the rate of cell death by increasing the rate of reoxygenation of hypoxic cells.

The late toxicity of a radiation treatment can be well described with the linear quadratic model applying dose fraction from 1 untill 8 Gy given intervals between dose applications longer than 6 hours. On the other hand for acute toxicity in early responding tissues as well as for neoplastic tissue this model can be applied only for the same total treatment time.

In dose fractionated radiotherapy the effect of different fractionation schedules could have substantial relevance in clinical practice.

According to the LQ formalism isoeffect curves calculating the effect of changing the dose fraction schedule and thus to compare two treatment regimens expressing an equal biological effect have been proposed [19]:

(3) $$\begin{array}{c} \frac{D_{\text{ref}}}{D_{\text{new}}}=\frac{\left(\alpha /\beta +d_{\text{new}}\right)}{\left(\alpha /\beta +d_{\text{ref}}\right)}; \end{array}$$

then

(4) $$\begin{array}{c} D_{\text{new}}=D_{\text{ref}}*\left[\frac{\left(\alpha /\beta +d_{\text{ref}}\right)}{\left(\alpha /\beta +d_{\text{new}}\right)}\right], \end{array}$$

where *D*_new_ is the new total dose obtained with a new fractionation schedule (*d*_new_) compared to a known total dose *D*_ref_ and fractionation dose (*d*_ref_) and the *α*/*β* ratio of the given tissue irradiated.

As proposed by Niranjan and coworkers [17] for single fraction irradiation the linear quadratic formula represents the probability of a desired outcome or undesired response (cure of a tumor or normal tissue injury) by the following probabilistic double exponential equation:

(5) $$\begin{array}{c} P\left(\text{response}\right)=E^{-[k*E^{-(\alpha *\text{dose}+\;\beta *{\text{dose}}^{2})}]}, \end{array}$$

where *P*(response) is the probability of cure or complications, EXP represents the number *e* (2.7183, commonly used in natural logarithms) risen exponentially to the power of the terms that follow, and *k* represents the number of clonogens in the target tissue, while *α* and *β* are coefficients for the respective linear and quadratic coefficients. The value of the *α*-coefficient divided by the *β*-coefficient (the *α*/*β* ratio) characterizes how a tissue or tumor is affected by different fractionation schemes.

The same group [17] proposed the following formula to equate the effect of a course of fractionated radiotherapy administered with dose *X* per fraction in terms of an equivalent dose for treatment with dose *Y* per fractions:

(6) [total  dose(x)]  ×  [1+X(α/β)] =  [total  dose(y)]  ×  [1+Y(α/β)],

where total dose(*y*) is the total dose given at *Y* Gy per fraction and is equal to the number of fractions times *Y*.

Formulas (1) and (2) reviewed allow drawing the following conclusions.

- The biological response of cell in terms of survival/death fraction exposed to a given radiation dose is expressed mathematically by a curve with linear (*α*) and exponential quadratic (*β*) component.
- The biological effect produced by ionizing radiation allows us to classify normal tissue in acute and early responding through defined *α*/*β* ratios expressed in Grays.
- The central nervous system is classified as late reacting tissue with different *α*/*β* ratios with regard to brain parenchyma (*α*/*β* = 2) and spinal cord (cervical 2-3—lumbar 4-5).

Formulas (3), (4), and (6) reviewed allow to calculate the response of a given tissue and relative *α*/*β* ratio to a different fractionation schedule.

Formula (5) defines the probabilistic effect of cell response when exposed to fractionated radiation.

This mathematic extrapolations derived by the LQ formula can be considered reliable when applied to dose fractionation. Nevertheless some remarks have to be made: first the LQ response formulas are not reliably applicable at any dose level [20, 21]. Furthermore some estimation of *α*/*β* ratios obtained with cell in vitro cultivated exposed to ionizing radiation was uncertain in particular of late responding tissue. Besides the LQ model does not take into consideration the time of radiation. To conclude uncertainty is reported in the literature about the *α*/*β* ratios of many tumors [17].

### 3.3. Controversies about Radiobiology of Radiosurgery for the Central Nervous System

Since its birth radiosurgery was a matter of debate among physicians. A number of contributions have been published since the early 90s describing all the possible issues that a conformal stereotactic single session radiation implies [16, 22, 23].

Several points have to be mentioned.

1. The CNS is classified as late responding tissue with accepted *α*/*β* ratio of ca. 2.
2. The delivery of a high focused radiation dose to a target implies a high conformality to produce a toxic effect limited to the target volume defined with a high steep dose falloff to avoid damage of the surrounding tissue.
3. The rationale of dose fractionation for radiation delivery to the CNS is to avoid the clinical consequences that a radionecrosis implies.

As previously reported in the literature [16] there was a consensus with the use of radiosurgery for benign brain lesions such as arteriovenous malformations (AVMs) and benign tumors, but its role for the management of malignant tumors is questioned. An improved ratio would be expected from fractionation for malignant lesions. The argument was based on the concept that hypoxic cells could reestablish their oxygenated state and become more sensitive to irradiation when treated with multiple fractions. Since AVMs and benign tumors are late-responding tissues, nothing was felt to be gained by fractionation.

Few months later, a reply to this contribution proposed a more detailed classification for the application of radiosurgery to the central nervous system with respect to target and surrounding tissues [23]:

1. late responding target embedded within late responding tissue: AVM,
2. late responding target surrounded by late responding tissue: meningioma/schwannoma,
3. early responding target embedded within late responding tissue: low gr. Glioma,
4. early responding target surrounded by late responding tissue: glioblastomas/metastases.

According to this oversimplified classification the indication to radiosurgery appears relatively simple to give. Nevertheless some variables must be considered: the different anatomic parts of central nervous system do not have the same uniform tolerance to radiation and 2nd cranial nerve optic chiasm and brainstem represent a dose limiting factor. The target volume plays a role to give a proper indication to radiosurgery. Finally the use of a highly conformal radiation dose is applicable with different techniques and the devices whose differences must be considered before applying radiosurgery.

*Reliability of the LQ Formalisms in Radiosurgery*. As previously reported in [24] the LQ model is simple and convenient, and by far it has been the most useful means for isodose calculation in treating tumors with conventional fractionated radiation therapy [24–27].

Despite the current use in clinical fractionated radiotherapy the LQ formalism has some limitations if applied to radiosurgery. First the LQ model cannot be applied with a dose of 8 Gy or more. Second the validity of the linear quadratic model has not been sufficiently investigated for very small target volumes (major diameter < 2 cm) so far. This implies that increasing the fractionation for slow growing tumors or nonneoplastic lesion may not bring a biological better response of the tumor if compared to a single dose if we accept a low *α*/*β* ratio for benign or slow growing tumors according to the most recent clinical data. Furthermore highly conformal radiosurgery spares dose exposure to surrounding tissue; therefore the compromise of dose fractionation to balance between imaging tumor control and normal surrounding tissue reaction might not be a consideration, depending on dose delivered, target volume, and eloquence of surrounding structures. To conclude many efforts to extrapolate a survival curve applying high doses to a benign lesions (>10 Gy) gave improbable values for which the risk of missing estimates of *α*/*β* ratios is too high, thus confirming the usefulness of such a model in radiosurgery [17, 24]. As previously published in the literature [17, 23] estimations of the equivalent dose for fractionated radiotherapy with 2 Gy fractions and single fraction radiosurgery doses using *α*/*β* = 2 commonly accepted for the brain as late reacting tissue are proposed.

The reliability of the LQ formalisms for high dose radiation is nowadays still strong matter of debate [28, 29]. A growing number of contributions analyzing laboratory and clinical show the risk of misestimating the equivalent dose by applying the LQ models for radiosurgery or high dose radiation fractions [6]. In his recent review Shibamoto and coworkers emphasize that many clinicians have used the LQ formalisms to convert hypofractionated doses to single doses [30–32]. Furthermore it is also reported that together with the introduction of the biological effective dose model (BED = *D*(1 + *d*/[*α*/*β*]) [19] the issue has become even more complicated thus not making the entire matter easier to understand.

Drawing outcomes from either laboratory data [33–39] and normal tissue data [25, 40–43] on of the author shows the reliability of LQ formalism if applied to isoeffects within a range of dose from 1–8 Gy. Nevertheless other authors proposed the validity of the LQ formalism and isoeffects curves also for doses higher than 8 Gy [28, 44]. Conversely to these theoretical extrapolations the same author reports further experimental data [45] which show contradictory results, in particular reliability of the LQ model for spinal cord as late reacting tissue with a fractionation dose up to 10 Gy.

Due to this uncertainty further mathematical models are also quoted: particular emphasis is given to the recently proposed survival curve model [46], the linear quadratic-linear model (LQL) [47, 48], and the generalized linear quadratic model (gLQ) [49]. Each of these models was developed modifying or existing model trying to draw conclusions which could better fit the isoeffect curves at high dose range; the experience with these models is somehow very limited and deserves further clinical application and laboratory lines of evidence to be used in clinical routine.

These contradictory findings are confirmed also by a previous report of the group from Pittsburgh [17] drawing outcomes from radiobiological analysis of clinical data.

1. The linear quadratic equation cannot reliably represent equivalent radiation effects when extrapolating from conventional fractionation (1.5–4 Gy per fraction) to high dose (12–25 Gy) single fractions for radiosurgery.
2. Mathematical models of radiation injury probability need to take into account that the target/tumor tissue's radiation response may affect the reaction of the surrounding normal tissue.
3. The predominant radiation response of a radiosurgical target is mediated through the target or tumor vasculature.

### 3.4. Experimental Lines of Evidence of Radiosurgery for Central Nervous System

The validity of the linear quadratic model for calculating isoeffect doses in radiation therapy has been intensively described [24, 26, 27, 50]. The model is based on the incidence of possible interactions of radiation of direct interactions of radiation with specific cellular targets (i.e., DNA strands). Because the LQ survival curve continuously bends downward with increasing radiation dose, some authors state that the LQ formula might overestimate cell death caused by high dose-per-fraction radiation therapy.

Conversely clinical results have shown that the LQ model actually underestimates tumor control by stereotactic radiation therapy or radiosurgery [29]. Therefore other mechanisms together with DNA strand breaks and/or chromosome aberrations may be involved in response of tumors to stereotactic radiation therapy or radiosurgery.

A number of experimental models have studied the effects of radiosurgery on the central nervous system [1, 5, 17]. The magnitude of radiosurgical effects remains poorly understood, especially when described in terms of conventional radiation therapy doses.

*Benign Tumors*. If we consider the application of radiosurgery for benign intracranial lesions like meningiomas and schwannomas, it has been observed that the radiobiological effect on meningiomas and other benign neoplasms is a combination of both cytotoxic and radiation induced vascular damage as already reported [5].

After both vestibular schwannoma and meningioma radiosurgery a doubling of the number of apoptotic cells after radiosurgery when compared to controls, within the first 48 h after irradiation, was observed.

*Metastases*. Some investigators have reported that apoptosis may play a significant role in the early effects of radiosurgery also for malignant tumors [51]. With regard to brain metastases some issues have to be considered. As previously reported [17] a RTOG dose escalation study (RTOG 90-05) [52, 53] showed results about dose tolerance of brain tissue after radiosurgery of recurrent brain metastases. A treatment protocol was defined applying initial dosis of 18, 15, and 12 Gy for diameters <20 mm, 21–30 mm, and 31–40 mm, respectively. A prescription dose escalation in 3 Gy steps was applied till toxicity rate was seen in over 30% of the sample population. The final recommendation was 24, 18, and 15 Gy for lesion of diameter <20, 21–30, and 31–40 mm, respectively. Furthermore it was reported that no difference in terms of progression free survival rate and tumor control was seen by stratifying the sample per histology of the brain metastases.

*Functional Radiosurgery*. An emerging field of radiosurgery is the treatment of functional neurological disorders which were interestingly the first clinical application evaluated once radiosurgery was introduced in the late 50s. After half a century we can describe an arising number of indications in the literature.

The first experiments about this issue were conducted with dose delivery of 150 Gy to small volumes producing tissue necrosis of the target (3 × 5 mm diameter within 1 month not changing over the ensuing years as reported by Kondziolka and coworkers) [3, 5, 54, 55]. Better outcomes in terms of desired targeting were observed with the use of 4 mm conic collimator. The doses applied were different with respect to the target volume and varied from 130 Gy for thalamotomies to 80 Gy for trigeminal neuralgia. As reported by Kondziolka and coworkers histological features of the target volumes after radiosurgery consisted in necrosis of the target volume a surrounding gliotic rim and normalisation of the parenchyma within 2 mm [56]. Even the higher doses used in functional radiosurgery do not appear to cause vascular injury to surrounding tissue with proper targeting, in accordance with the findings of more recent studies [57].

*The Issue of Vascular Damage in Radiosurgery*. It is well known that the radiobiological effect of radiosurgery is based on direct cytotoxic effect after low dose radiation therapy [58].

On the other hand it is well accepted that intratumor microenvironment greatly influences the radiosensitivity of tumor cells and the intratumor microenvironment is closely related to the functional status of tumor microvasculature [57].

Available information from AVM radiosurgery or meningioma radiosurgery has shown that normal vessels rarely decrease in size or occlude after radiosurgery and therefore they conclude that the abnormal vessels of neoplasms or vascular malformations have a relative sensitivity to radiosurgery in comparison to normal surrounding vessels since no occurrence of perforator occlusion leading to an infarct has been identified [5]. On the other hand it must also be said that chance to produce a damage of normal capillary vessels is directly proportional to the dose increasing [59].

In a recent review of the literature [57] an analysis of the studies published about vascular damage in tumors after stereotactic high dose hypofractionated radiation therapy and radiosurgery was performed. The authors indicate that the functional vascularity in human tumors remains unchanged or improves slightly during the early period of conventional fractionated radiotherapy with 1.5–2.0 Gy daily doses but gradually diminishes during the latter part of treatment. By delivering radiation doses higher than 10 Gy in a single fraction or 20–60 Gy in limited numbers of fractions severe vascular damage leading to the deterioration of the intratumor microenvironment and indirect death of tumor cells is observed. Of note experimental data about radiation induced vascular damage shows that high dose delivery in single session produces decrease of vascular volume and increase of vascular permeability. It is also observed that radiation induced changes in blood perfusion, functional intravascular volume, and vascular permeability are directly related to the functional integrity and activity of endothelial cells. The authors strictly distinguished between endothelial cells derived from normal and tumor tissue classifying them as radioresistant and radiosensitive, respectively, in accordance with other experimental lines of evidence [60] demonstrating that developing vessels are more radiosensitive than mature vessels.

Most specifically as reported by the authors [57] the death of endothelial cells after direct radiation damage would cause focal microscopic or macroscopic vascular damage and collapse of the affected capillary-like vessels. Soon after vascular permeability in tumors increases rapidly after irradiation due to damage in the endothelial cells followed by widening of the gaps between endothelial cells. Further extravasation of plasma due to vascular permeability might increase the erythrocyte concentration within the narrow capillaries, thereby leading to retardation or stasis of blood perfusion. In addition, the increased permeability of capillaries may increase the extravascular or interstitial plasma protein concentrations, thereby elevating interstitial fluid pressure. The elevation of interstitial fluid pressure above the intravascular blood pressure will cause vascular collapse. Therefore, it is probable that the early decline in functional vascularity after irradiation in tumors may be caused at least in part by collapse of blood vessels as a result of elevation of interstitial fluid pressure. When tumor volume shrinks due to death of parenchymal cells after irradiation, the tumor vascular beds may become further disorganized, aggregated, and fragmented.

The authors concluded that the radiation-induced vascular damage and the resulting indirect death of tumor cells play important roles in the response of tumors to high dose hypofractionated radiotherapy and radiosurgery. In addition, enhanced immune reactions and increased eradiation of cancer stem cells might be involved in the response of tumors to stereotactic fractionated radiotherapy and radiosurgery [57].

*Toxicity of Radiosurgery*. These emerging applications of radiosurgery open as the last point the discussion about the different dose tolerance of brain structures after radiosurgery. With regard to radiobiological properties the CNS is a “late reacting tissue.” Despite this not all brain regions including cranial nerves have the same eloquence.

The incidence of cranial neuropathies after radiosurgery is well described in the literature. Variables to consider are the dose delivered and its distribution (isodose line), the volume of the tissue irradiated, the sensitivity of the tissue affected, and history of any prior irradiation. The clinical manifestation may vary greatly according to the volume and location of the target [13]. As previously reported [61], a prior history of fractionated radiation therapy to the same region of interest appears to have limited effects on the risk of developing postradiosurgery parenchymal edema with exception to the optic nerve. Each cranial nerve has a specific tolerance to dose radiation. According to clinical experience with radiosurgery and conventional fractionated radiotherapy sensory nerves appear to be the most sensitive, followed by somatic sensory nerves and motor nerves [61]. The anterior optic pathways are the most dose sensitive structures, thus implying that the dose applying the second cranial nerve (optic nerve) should be always a consideration. Many contributions report a dose maximum tolerance of the optic nerve after radiosurgery to be at 8 Gy. Nevertheless more recent studies [62] report that the maximal dose tolerance at 10 Gy may be related to better imaging technique available. The correlation of data with dose fractionation to conclude brought physicians to assume an *α*/*β* ratio for the optic nerve of ca. 1 as reference value to calculate dose equivalent between fractionation and radiosurgery dose protocols.

Furthermore the clinical experience with AVM radiosurgery allowed also defining specific dose tolerance within the brain parenchyma. Results of an AVM radiosurgery study group tolerance showed no difference in the likelihood of injury imaging changes after radiosurgery for AVMs with respect to brain location. Conversely relevant differences were seen in the development of clinical side effects being then observed at the frontal region as the most radioresistant followed by parietal region, temporal lobe, cerebellar and brainstem and thalamus/basal ganglia as the most sensitive [61, 63, 64]. 

*The Issue of Volume Dose Effect*. A major issue in radiosurgery for CNS is not only the highly conformal dosis to deliver in order to achieve in one session the same radiobiological effect of a dose fractionation schedule but also the effect of the target volume. According to experimental data [65] the tolerance of the spinal cord of rats increases from 20 Gy to 80 Gy delivered in single session if the volume of spinal cord irradiated is reduced from 20 mm to 2 mm.

For target volumes with diameter more than 3 cm the dosis falloff at the target margin is progressively flatter thus increasing the volume of normal tissue irradiated and then the toxicity rate. Better appreciation of the tolerance of CNS structures to high radiation dose schedules is still a matter of investigation and requires further studies.

## 4. Conclusions

The radiobiology of radiosurgery in particular for the central nervous system plays a crucial role for the clinical indication and the application of a therapeutic radiation dose in single session. The current mathematical models applied for dose fractionation allow us to predict the rate of late neurological side effects but are not applicable for a dose over 8 Gy. Therefore in order to avoid late complications a highly conformal radiation delivery with high dose gradient is required. Target location and volume are crucial to achieve the conformality required. The current indications to radiosurgery concern small rest or recurrent benign lesions and metastases and in the last years also for small imaging defined benign lesions without histological confirmation. Radiosurgery for functional neurological diseases is an emerging field and the results are encouraging. As previously reported [33] the effects of radiosurgery on the brain tumor microenvironment are still under investigation. The pathogenesis of biological effects after radiosurgery may be unique. The need for basic research concerning the radiobiological effects of high dose, single fraction, and ionizing radiation on nervous system tissue is crucial in particular to define mathematical models reliable to this special radiation technique. Particular emphasis should be given to the mechanisms of vascular damage after high dose radiation and the tolerance doses of CNS structures for radiosurgery.
